# Supplementary material for: A sequential three-way decision model based on linguistic Z-numbers
Source: PLoS One. 2025 May 5;20(5):e0320350. doi: 10.1371/journal.pone.0320350 (PMC12052160; doi:10.1371/journal.pone.0320350)
Supplement: Attachment — Submitted filename: Respone to Reviewers.pdf [file pone.0320350.s001.pdf]

Dear Dr. Chu and Reviewers,

Thank you very much for your valuable feedback and for taking the time to review our manuscript. We greatly appreciate the thoughtful comments and suggestions provided, which have helped to improve the quality of our work. We have carefully considered each point raised by the reviewers and the editor, and we have made revisions accordingly to address the concerns and strengthen the manuscript. Below is a summary of the major revisions made in response to the reviewers' comments:

1 Respone to Editor Comments

- **Comment 1:** Please ensure that your manuscript meets PLOS ONE's style requirements, including those for file naming.

- **Response:** We sincerely appreciate the editorial team's guidance on adhering to PLOS ONE's style requirements. We have carefully reviewed the PLOS ONE style templates provided at the links and have revised our manuscript accordingly. Specifically: 1. Headings and Subheadings: We have ensured that all headings and subheadings are written in sentence case, capitalizing only the first word, proper nouns, and genus names, as per the style guidelines. 2. Figure and Table Titles: The titles of all figures and tables have been revised to align with PLOS ONE formatting requirements. We also checked that all figures and tables are appropriately cited in the main text. 3. Other Formatting Requirements: We have reviewed and updated the manuscript to ensure it meets additional formatting requirements, such as consistent font usage, proper spacing, and alignment of text and figures. To ensure transparency, all changes made in response to this comment are marked in blue in the revised manuscript. Thank you for providing these helpful resources, which greatly aided in improving the presentation of our work.

- **Comment 2:** We suggest you thoroughly copyedit your manuscript for language usage, spelling, and grammar. If you do not know anyone who can help you do this, you may wish to consider employing a professional scientific editing service. The American Journal Experts (AJE) (https://www.aje.com/) is one such service that has extensive experience helping authors meet PLOS guidelines and can provide language editing, translation, manuscript formatting, and figure formatting to ensure your manuscript meets our submission guidelines. Please note that having the manuscript copyedited by AJE or any other editing services does not guarantee selection for peer review or acceptance for publication.

- **Response:** We appreciate the suggestion to thoroughly review the manuscript for language usage, spelling, and grammar. In response to this comment, we have carefully copyedited the manuscript to ensure clarity, accuracy, and compliance with PLOS ONE's language requirements. Additionally, we have provided the following files with our resubmission: 1. Revised Manuscript with Track Changes: A version of the manuscript highlighting all changes made during the revision process (uploaded as a supporting information file). 2. Clean Copy of the Manuscript: A final version of the manuscript without tracked changes (uploaded as the new manuscript file). All revisions were conducted internally, and we made every effort to ensure the

manuscript adheres to the highest standards of language and formatting. Thank you for emphasizing the importance of this aspect in the submission process.

- **Comment 3:** We note that your Data Availability Statement is currently as follows: "All relevant data are within the manuscript and its Supporting Information files." Please confirm at this time whether or not your submission contains all raw data required to replicate the results of your study. Authors must share the "minimal data set" for their submission. PLOS defines the minimal data set to consist of the data required to replicate all study findings reported in the article, as well as related metadata and methods.

- **Response:** We thank the editorial team for their valuable feedback on data availability. We confirm that all data required to replicate the results of our study are included within the manuscript and its Supporting Information files. Specifically, the initial data used in this study are presented in Tables 3 and 4, which serve as the foundation for our analysis. The conclusions of the manuscript can be fully reproduced by applying the proposed model to the data provided in these tables. We appreciate the opportunity to clarify this and remain committed to ensuring transparency and reproducibility in our research.

- **Comment 4:** PLOS requires an ORCID iD for the corresponding author in Editorial Manager on papers submitted after December 6th, 2016. Please ensure that you have an ORCID iD and that it is validated in Editorial Manager. To do this, go to 'Update my Information' (in the upper left-hand corner of the main menu), and click on the Fetch/Validate link next to the ORCID field. This will take you to the ORCID site and allow you to create a new iD or authenticate a pre-existing iD in Editorial Manager.

- **Response:** Thank you for the reminder regarding the ORCID iD requirement. We have ensured that the corresponding author's ORCID iD is registered and successfully validated in the Editorial Manager system. We appreciate your guidance in maintaining compliance with PLOS ONE's submission requirements.

- **Comment 5:** Please update your submission to use the PLOS LaTeX template. The template and more information on our requirements for LaTeX submissions can be found at http://journals.plos.org/plosone/s/latex.

- **Response:** Thank you for the reminder regarding the PLOS LaTeX template. We have revised our manuscript using the latest PLOS LaTeX template provided on the journal's website. All formatting and style requirements have been carefully implemented to ensure compliance with the submission guidelines.

- **Comment 6:** We note you have included a table to which you do not refer in the text of your manuscript. Please ensure that you refer to Table 2 in your text; if accepted, production will need this reference to link the reader to the Table.

- **Response:** We apologize for any misunderstanding caused by the previous version of the manuscript regarding Table 2. Table 2 represents our Z-cost parameters output, and we have now updated the manuscript to properly reference it within **Assumption 1**, which states: Assumption 1: Both the attribute values and cost

parameter values are linguistic Z-numbers. The Z-cost parameters table is indicated by Table 2. Thank you for bringing this to our attention, and we appreciate your careful review of our submission.

- **Additional Editor Comment 1:** Please consider the following suggestions to revise your work for possible publication. The introduction is too long. Introduction and literature review should be separated into two independent sections. The introduction is to precisely present the research's background, purpose, the advantages of the proposed method compared to the existing papers, findings of comparisons, and paper structure, etc. A more in-depth literature review of relevant methods, including LZs, HULZNs, HUDLZNs, LZFSs, PROMETHEE, PROMETHEEII, HULZPWA, HULZPWG, LZOWGA, TODIM, VIKOR, TWDM, etc., should be conducted to clearly show the research gap that needs to be addressed; for example, TWDM related methods should be briefly introduced.

- **Response:** Thank you very much for your valuable suggestions. We greatly appreciate your insightful comments, which have helped us improve our manuscript. We carefully reviewed many research papers published in your esteemed journal and found that it is uncommon to separate the literature review into a standalone section. Therefore, we have decided to keep the introduction and literature review in one section to maintain consistency with the journal's typical style. However, following your suggestion, we have significantly refined the introduction to make it more concise. The revised introduction now clearly presents the research background, purpose, advantages of the proposed method, and the paper structure. Additionally, we have expanded the literature review to include a more in-depth discussion of relevant methods, such as LZs, HULZNs, HUDLZNs, LZFSs, PROMETHEE, PROMETHEEII, HULZPWA, HULZPWG, LZOWGA, TODIM, VIKOR, TWDM, etc., to highlight the research gap that this study aims to address. We have also briefly introduced TWDM-related methods as requested. We hope this approach balances your recommendations with the journal's style. Detailed revisions have been marked in blue in the revised manuscript for your review. Thank you again for your constructive feedback.

- **Additional Editor Comment 2:** In section 6, a comparison with one method, i.e., LZOWGA aggregation operator-based method, is insufficient. A comparison with more methods needs to be made to display the effectiveness of the proposed method. Similarly, more methods, other than only VIKOR, need to compared.

- **Response:**Thank you for your professional suggestions. We greatly appreciate your insightful comments regarding the need for a broader comparison with other methods. After carefully reviewing relevant literature, we found that methods for handling decision-making problems in the LZs environment can be categorized into three main types: 1. Aggregation Operator Methods: These utilize specific operators, such as the LZOWGA operator used for comparison in our study. 2. Integration with Classical Decision-Making Methods: For instance, the integration with VIKOR, which is also included in our analysis. 3. Language Scale Function (LSF) Methods: These involve the use of linguistic scale functions to address LZs-related problems. In response to your comments and to enhance the comprehensiveness of our work, we have included a sensitivity analysis based on LSF methods in

the revised manuscript(Section6). This addition provides further insights into the robustness and effectiveness of our proposed method compared to alternative approaches. All changes related to this enhancement have been highlighted in blue in the revised manuscript for your reference. Thank you again for your valuable feedback, which has significantly improved the depth and rigor of our study.

- **Additional Editor Comment 3:** The most recently cited paper was published in 2022, which is old and should be updated to 2024.
- **Response:**Thank you for pointing out this issue regarding the references. We have carefully reviewed and updated our reference list to include more recent studies published up to 2024, ensuring that our work reflects the latest research trends. At the same time, we have retained references that are closely related to our study to provide essential foundational knowledge and context. We appreciate your suggestion, as it has helped us improve the relevance and quality of our manuscript.

2 Respone to Reviewer #1 Comments

- **Comment 1:** Overall, paper is well written. The method is well explained. However, the motivation on why the suggested method necessary is vague, not explained.
- **Response:** Thank you for pointing out the lack of clarity in explaining the motivation behind the suggested method. In the revised manuscript, we have provided a more detailed explanation of why the proposed method is necessary. Specifically, we have emphasized the limitations of existing methods in addressing decision-making problems under LZs environments and how our approach overcomes these challenges. The revisions have been highlighted in red for your review.
- **Comment 2:** Page 13: Bring Step 1 to the next paragraph.
- **Response:** We apologize for any confusion caused by the original placement of Step 1. Following your suggestion, we have moved Step 1 to the next paragraph on page 13. This adjustment improves the logical flow and ensures the text is easier to follow.
- **Comment 3:** Fig.2 & Fig.3 exactly the same figure. If they are the similar figure, just show once and recall the figure for other sections.
- **Response:** We sincerely apologize for the oversight in duplicating Figures 2 and 3. After reviewing the content, we have removed the redundant figure and retained only one. The manuscript now references the single figure appropriately in all relevant sections, avoiding any unnecessary repetition.

Thank you again for your invaluable feedback, which has significantly improved the quality of our work.
